# Globally shared TCR repertoires within the tumor-infiltrating lymphocytes of patients with metastatic gynecologic cancer

**DOI:** 10.1038/s41598-023-47740-2

**Published:** 2023-11-22

**Authors:** Taehoon Kim, Hyeonseob Lim, Soyeong Jun, Junsik Park, Dongin Lee, Ji Hyun Lee, Jung-Yun Lee, Duhee Bang

**Affiliations:** 1 Department of Chemistry, Yonsei University, 50 Yonsei-ro, Seodaemun-gu, Seoul, 03722 Korea; 2 Department of Obstetrics and Gynecology, Yonsei University, 50 Yonsei-ro, Seodaemun-gu, Seoul, 03722 Korea; 3 Department of Clinical Pharmacology and Therapeutics, College of Medicine, Kyung Hee University, 26 Kyungheedae-ro, Dongdaemun-gu, Seoul, 02447 Korea; 4 Department of Biomedical Science and Technology, Kyung Hee Medical Science Research Institute, Kyung Hee University, 26 Kyungheedae-ro, Dongdaemun-gu, Seoul, 02447 Korea

**Keywords:** Cancer, Computational biology and bioinformatics, Immunology, Diseases, Medical research

## Abstract

Gynecologic cancer, including ovarian cancer and endometrial cancer, is characterized by morphological and molecular heterogeneity. Germline and somatic testing are available for patients to screen for pathogenic variants in genes such as BRCA1/2. Tissue expression levels of immunogenomic markers such as PD-L1 are also being used in clinical research. The basic therapeutic approach to gynecologic cancer combines surgery with chemotherapy. Immunotherapy, while not yet a mainstream treatment for gynecologic cancers, is advancing, with Dostarlimab recently receiving approval as a treatment for endometrial cancer. The goal remains to harness stimulated immune cells in the bloodstream to eradicate multiple metastases, a feat currently deemed challenging in a typical clinical setting. For the discovery of novel immunotherapy-based tumor targets, tumor-infiltrating lymphocytes (TILs) give a key insight on tumor-related immune activities by providing T cell receptor (TCR) sequences. Understanding the TCR repertoires of TILs in metastatic tissues and the circulation is important from an immunotherapy standpoint, as a subset of T cells in the blood have the potential to help kill tumor cells. To explore the relationship between distant tissue biopsy regions and blood circulation, we investigated the TCR beta chain (TCRβ) in bulk tumor and matched blood samples from 39 patients with gynecologic cancer. We found that the TCR clones of TILs at different tumor sites were globally shared within patients and had high overlap with the TCR clones in peripheral blood.

## Introduction

Gynecologic cancers, including ovarian cancer and endometrial cancer, cause widespread mortality among women^1,2^. Morphological and molecular heterogeneity in gynecologic cancers has been shown to affect patient survival^3,4^. Pathologic assessment of genomic (e.g. TP53, PTEN, and BRCA1/2) and immunogenomic (e.g. PD-1 and PD-L1) features to distinguish diverse subtypes of gynecologic cancer is important to determine optimal treatment strategies^5–13^. In this context, the approval of Dostarlimab is noteworthy, as it specifically targets patients with mismatch repair deficiency (dMMR) or microsatellite instability-high (MSI-H) markers^14^. The US Food and Drug Administration (FDA) granted accelerated approval to Dostarlimab for monotherapy use in patients with mismatch repair deficiency (dMMR) in 2021^15^. The approval was further extended in 2023, allowing the use of Dostarlimab for recurrent or advanced endometrial cancer patients with either mismatch repair deficiency (dMMR) or microsatellite instability-high (MSI-H), both in combination with chemotherapy and as monotherapy^16,17^. Additionally, the FDA granted approval to Pembrolizumab for patients with persistent, recurrent or metastatic cervical cancer whose tissue expresses PD-L1 (CPS ≥ 1) in 2021^18,19^. This regulatory endorsement underscores the importance of genomic and immunogenomic profiling in tailoring treatment approaches for gynecologic cancers.

The success of immune checkpoint inhibitors has led to heightened interest in the tumor immune microenvironment as a factor in the diagnosis and treatment of gynecologic cancers^20–22^. Investigation of TCR repertoire which is expressed by heterogeneous lymphocyte populations is important for understanding the immune activities surrounding tumors. Previous studies have shown evidence of T cell expansion with TCR repertoire or flow cytometry data of TILs in gynecologic cancers depending on the subtype of the tumor tissue^23,24^ and T cells^25^. However, the number of tissue samples per patient was not high enough in these studies to assert that the TCRs found in tissues are highly individual-specific. Also, an explanation of the TCR found in multiple tissues and its overlap with blood was insufficient.

**Figure 1:**
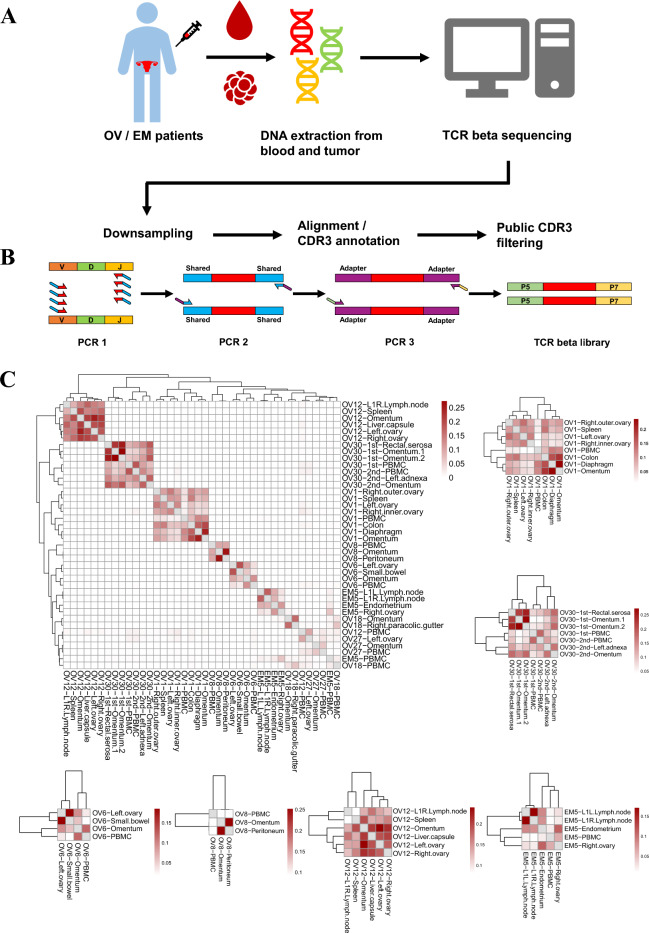
TCR clonal similarity between patient samples. (**A**) Scheme of the workflow in the study. PBMCs were separated from patients’ whole blood collected prior to tumor resection. Extracted DNA from paired PBMC and tissue samples was subjected to TCRβ library preparation. FASTQ files were subsampled to a size of 1 Gb. CDR3 sequences were excluded if they matched with downloaded public CDR3 sequences (see Methods). (**B**) A brief summary of the TCRβ library preparation procedures (see Methods). (**C**) Pairwise overlap was calculated and applied to hierarchical clustering (see Methods). Cases labeled as “OV30-1st” and “OV30-2nd” were from a single patient that underwent surgery twice. Six representative insets of patients with metastasis are followed. CDR3s observed in both replicates were termed twice-observed CDR3 segments, while those observed in only one replicate were referred to as once-observed CDR3 segments. In (**C**), only the twice-observed CDR3 segments were used for the pair-wise overlap analysis.

In this study, we sought to find patterns in inter-sample TCR repertoire overlap which consists of overlap between tissue-tissue and tissue-blood. We performed sequencing of the TCRβ from both tumor and peripheral blood samples of 31 patients with ovarian cancer and 8 patients with endometrial cancer. We also examined multiple metastatic tumor sites in 8 patients and compared to the primary tumor. Experiments were performed in duplicate to focus on clones that were twice-observed in both replicates of each sample. The overall scheme is shown in Fig. 1A,B. Our work highlights the possibility of using patient peripheral blood samples to discover TCR clones that are present among TILs distributed across multiple tissue sites.

## Methods

### Subjects

Thirty-nine patients who underwent surgery in 2020 or 2021 in the Department of Obstetrics and Gynecology at Yonsei University, Korea, participated in the study. Institutional Review Board (IRB) approval was obtained from Yonsei University Health System (IRB number 4-2018-0342). All methods were performed in accordance with relevant guidelines and regulations. All patients gave informed consent to participate in the study. Tissue samples were collected from 31 patients with ovarian cancer and 8 patients with endometrial cancer. The 33 cases from 31 ovarian cancer patients included borderline ovarian tumor and peritoneal cancer (ICD-O codes: D39.1, C48.1, C48.2). Whole blood samples were collected from each patient prior to tumor resection. The patient labeled “OV30” and “OV31” had serial samples. The first series of samples were obtained for the purpose of pathologic confirmation and the second series of samples were obtained while reducing the size of the tumor.

### Clinical sample processing

Peripheral blood mononuclear cells (PBMCs) were isolated from whole blood samples by a density gradient method using Ficoll-Paque PLUS^®^ (GE Healthcare). DNA was extracted from tissue and PBMC samples using DNeasy Blood & Tissue Kits^®^ (Qiagen). The concentration of DNA was measured using a Qubit dsDNA BR Assay Kit^®^ (Invitrogen). Twenty microliters of extracted DNA were used for each experimental replicate, with input DNA ranging from 94 to 4240 ng.

### TCRβ library preparation

Genomic DNA from tissues and PBMCs was amplified by three steps of multiplex PCR. Before library preparation, primer rebalancing with an oligo pool containing primer binding sites and random barcodes was conducted to prevent amplification bias. In the first PCR, the forward primer targeted the V region upstream of complementarity-determining region 3 (CDR3) in TCRβ, and the reverse primer targeted the J region of TCRβ. The primer sequences used in the first PCR contained shared flanking sequences, which enabled the second PCR to attach Illumina sequencing adapters (Supplementary Table S4). The first PCR was performed with 20 μL template DNA, 5 μL each of forward and reverse primer mix (100 μM), 10 μL 5X Q solution, and 25 μL 2X Qiagen Multiplex PCR Master Mix^®^ (Qiagen). The first PCR was performed with the following conditions: 95 °C for 10 min, 25 cycles of 95 °C for 30 s, 58 °C for 90 s, and 72 °C for 90 s, and a final extension at 72 °C for 3 min. The PCR products were purified using 1.5X AMPure XP beads (Beckman Coulter) and eluted in 12 μL nuclease-free water.

The second PCR was performed with 12 μL of the first PCR product, 2.5 μL each of the forward and reverse primer mix (10 μM), 10 μL 5X Q solution, and 25 μL 2X Qiagen Multiplex PCR Master Mix^®^ (Qiagen). The second PCR was performed with the following conditions: 95 °C for 15 min, 6 cycles of 95 °C for 30 s, 60 °C for 40 s, and 72 °C for 1 min, and a final extension at 72 °C for 10 min. The PCR products were purified using 1.5X AMPure XP beads and eluted in 22 μL nuclease-free water.

The third PCR was performed with 22 μL of the second PCR product, 2.5 μL each of the forward and reverse primer mix (10 μM), and 25 μL 2X KAPA Hifi PCR^®^ solution (KAPA Biosystems). The third PCR was performed with the following conditions: 98 °C for 3 min, 6 cycles of 98 °C for 10 s, 65 °C for 30 s, and 72 °C for 30 s, and a final extension at 72 °C for 5 min. The PCR products were purified using 1.2X AMPure XP beads and eluted in 22 μL nuclease-free water. The concentration of the final PCR product was measured using a Qubit dsDNA BR Assay Kit^®^ (Invitrogen).

### Sequencing and data processing

The library was sequenced as 2 × 150 bp paired-end readout with NovaSeq 6000 System^®^ (illumina). The fastq files were randomly subsampled to a size of 1 Gb using Seqtk (version 1.3-r106). CDR3 sequences were called by MiXCR (version 3.0.13)^26^ using the following options: -s Homosapiens –starting-material dna –adapters adapters-present –impute-germline-on-export –5-end v-primers –3-end j-primers –receptor-type trb –region-of-interest CDR3 –only-productive –align "-OvParameters.geneFeatureToAlign = VRegion" –assemble "-OaddReadsCountOnClustering = true" –verbose.

Clones with non-human epitopes were filtered out by a screen against 7,276,705 CDR3 amino acid sequences downloaded from TCRdb^27^, McPAS-TCR^28^, PIRD^29^, TCR3d^30^, and VDJdb^31,32^. Some clones were excluded if junction sequence of the clone does not contain proper conserved residues (e.g. V-Cystein at 104th or J-Phenylalanine at 118th to 129th). The average number of unique CDR3 amino acid sequences for each replicate in patients was 12,917.4, ranging from 2984.3 to 82,681.7.

### TCR repertoire analysis

**Figure 2:**
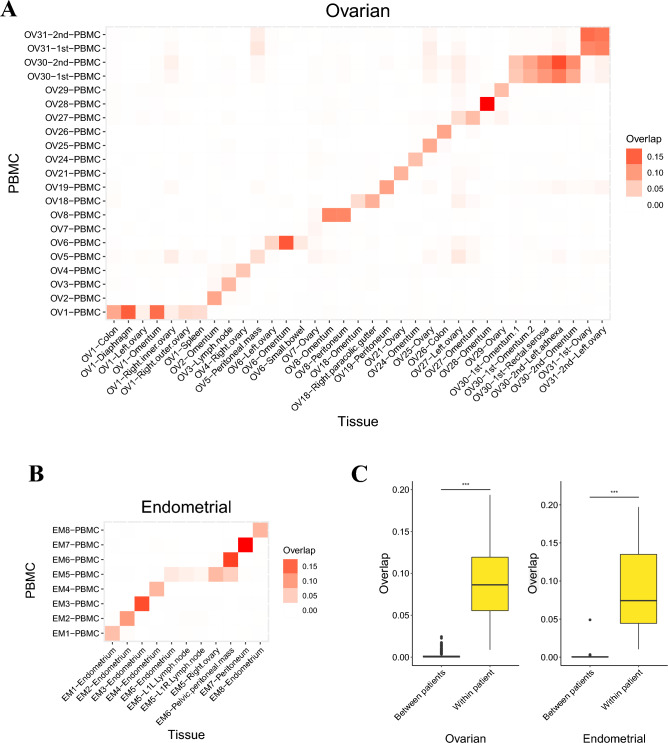
Overlap between TIL and PBMC TCR repertoires is patient specific. (**A**) Overlap in TCR repertoires between PBMCs and ovarian cancer tissue samples. Two patients who each underwent surgery at two different time points are included (“OV30-1st” and “OV30-2nd”, “OV31-1st” and “OV31-2nd”). (**B**) Overlap in TCR repertoires between PBMCs and endometrial cancer tissue samples. For both (**A**) and (**B**), only the twice-observed CDR3 segments were utilized for the overlap analysis. (**C**) Comparison of overlap between patients and within patients. “Between patients” refers to sample pairs from different patients. “Within patients” refers to sample pairs from the same patient, which includes the sample pairs from different time points. Wilcoxon rank-sum test, $${}^{***}p$$<0.005.

**Figure 3:**
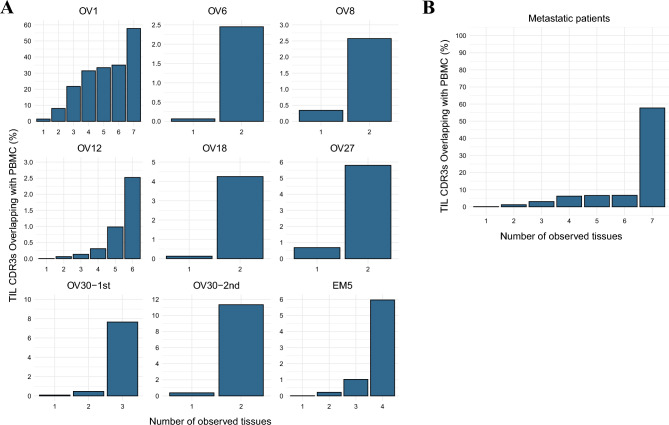
The PBMC TCR repertoire has high overlap with clones found in multiple tissues with metastasis. (**A**) In patients with metastatic cancer, unique tissue CDR3 segments were grouped based on the number of tissues in which they were detected. The proportion of these unique tissue CDR3 segments also found in the patients’ PBMCs is represented as a percentage. (**B**) The proportions displayed in (**B**) are based on the cumulative counts of observed tissue CDR3 segments and those TIL CDR3s overlapping with PBMCs for all 9 cases, rather than averaging individual data. Merged tissue replicates refer to the union of CDR3 segments from both replicates, including once-observed CDR3 segments. In analyses (**A**) and (**B**), the TIL TCR repertoire was derived from these replicates to evaluate clonal overlap across multiple tissues. Conversely, the PBMC TCR repertoire was confined to twice-observed CDR3 segments, enhancing the reliability of comparisons with TIL TCR repertoires. This approach accounts for the diversity and clonal expansion within the PBMC repertoire, providing a comprehensive quantitative assessment.

The term “segment” was used to refer to functionally annotated sequences in TCRβ. Unique CDR3 segments were defined as unique CDR3 amino acid sequences. The frequency of each clone was calculated as the number of reads spanning a unique CDR3 segment over the total number of reads spanning all CDR3 segments. The abundance of each group was calculated as the total number of reads within a group divided by the total number of reads in the sample, which is equal to the sum of the frequencies of all clones in the group. Unique CDR3 segments observed in both replicates were considered as twice-observed CDR3 segments, while those observed in only one replicate were considered once-observed CDR3 segments. Only twice-observed CDR3 segments were used for the calculation of inter-sample overlap (Figs. 1C and 2A,B). Merged tissue replicates, which also contained once-observed CDR3 segments, were used to observe clones overlapping multiple tissues (Fig. 3A,B).

The sample-wise TCR repertoire overlap was determined by the “repOverlap” function in the R immunarch package using the “jaccard” and “morisita” arguments^33^. Graphs were generated using base R (https://www.r-project.org/) and the ggplot2 package^34^. The ggsignif package was used to annotate the plots with statistical significance levels. The reshape2 package was used to format data to generate heatmaps^35^. The viridis package was used to assign colors in the plots.

The term “motif” was used to refer to a unit of local or global similarity in CDR3 amino acid sequences observed as a k-mer. TCRβ CDR3 clustering was performed with GLIPH2 (version 0.01)^36^ using CD8 reference sequences and the default parameters. Replicates were merged to capture more clusters with CDR3 sequence similarity. Because each patient’s HLA type is unknown, tissue samples from different patients were clustered separately. If separate clusters of CDR3 sequences within a patient contained overlapping motifs, the cluster with the lowest Fisher score was retained, and the other clusters were removed. The motif frequency was calculated by dividing the total number of reads spanning an individual motif by the total number of reads in the sample.

Shannon’s entropy was gauged by following equation:

$$Shanno{n}^{\mathrm{^{\prime}}}s entropy=-\sum_{i=1}^{n}{p}_{i}{log}_{2}{p}_{i}$$

where n is the total number of unique clones in the TCR repertoire, and ${p}_{i}$ is the frequency of one unique clone in the TCR repertoire. Shannon’s entropy was used as a measure for repertoire diversity.

### Statistical analysis

All statistical tests were performed in R. Wilcoxon rank-sum test and Wilcoxon signed-rank test were performed to assess significance in comparisons between groups. The Bonferroni method was applied to correct errors due to multiple comparisons. The Chi-squared test was used to calculate the *p*-value in Supplementary Table S2.

### Target-enriched library preparation and variant calling

Genomic DNA from tumor tissues and PBMCs were sheared using covaris (Covaris), and 50–100 ng sheared genomic DNA was used for tumor variant analysis. End repair and A-tailing of sheared genomic DNA were performed using 5 × ER/A-Tailing Enzyme Mix (Enzymatics), and adaptor ligation was performed using WGS Ligase (Enzymatics). Adaptor-ligated genomic DNA was purified with 1.2 × AMPure XP beads and eluted in 20 μL nuclease-free water. PCR amplification was performed with the following steps: 3 min at 98 °C; 10 cycles of 15 s at 98 °C, 30 s at 60 °C, and 30 s at 72 °C; followed by 10 min at 72 °C. The PCR amplicons were purified using 1.2 × AMPure XP beads and analyzed using the 4150 Tapestation system (Agilent). Target enrichment was performed using the AlphaLiquid^®^ 100 target-capture panel, which consists of 106 cancer-related genes^37^. Target-enriched DNA libraries were sequenced using an Illumina NovaSeq 6000 System^®^ to create 150 bp paired-end reads.

Adaptor sequences and reads with low quality (< Q20) were trimmed using FASTP^38^. The trimmed reads were aligned to the human reference genome (hg38) using the BWA “mem” algorithm^39^. Then, duplicate reads were removed, and variants were called using VarDict^40^. Variants of tumor tissues from the same patient were combined, and germline variants were removed. For each patient, variants with allele frequency > 1% in at least one tumor tissue were retained, and other variants were removed.

## Results

### Similarity landscape of TCR repertoire across all gynecologic cancer patients

The median age of the study population was 57 years (range 17–86 years). Nine patients (23.1%) had a history of hypertension, and four patients (10.3%) had diabetes mellitus. One of the patients with endometrial cancer had a history of colon cancer, and another had a history of thyroid cancer. Among the patients with epithelial ovarian cancer (n = 27), most presented with high-grade serous carcinoma (n = 20, 74.1%) and stage III–IV disease (n = 25, 92.6%), and eight (29.6%) had BRCA1/2 mutation.

To analyze the TCR repertoire, we generated Illumina sequencing libraries from genomic DNA isolates of the samples by PCR reactions using primers targeting TRBV and TRBJ genes (see Methods). We assumed that unique CDR3 segment represents TCR clone since CDR3 of TCRβ is highly diverse due to VDJ recombination. In the process of filtering, clones associated with non-human epitopes were screened against a database consisting of 7,276,705 CDR3 amino acid sequences sourced from TCRdb^27^, McPAS-TCR^28^, PIRD^29^, TCR3d^30^, and VDJdb^31,32^. Precisely, clones were excluded based on an exact match with sequences from these databases. Non-human epitope CDR3 was removed to rule out the TCR repertoire that are unrelated to tumor antigen, such as TCR repertoires that are expanded by pathogens. Consequently, an average of 6,130.6 clonotypes, which cumulatively constituted about 29.6% frequency, were removed per sample. Among the CDR3 segments that passed the filtering process, twice-observed CDR3s showed higher frequencies compared to once-observed CDR3s (Methods, Fig. S1). The frequency of twice-observed CDR3 segment was consistent in both replicates (Fig. S2), so we thought that twice-observed CDR3 segments possibly represent expanded clones.

We assessed the pairwise similarity in TCR repertoire between all possible pairs of samples by hierarchical clustering and Jaccard index calculation^41^ to see if there were shared clones. The Jaccard indices were zero in most cases which means no overlap is observed between different patients (Fig. 1C). This trend is consistent with previous articles^42,43^. We assumed that many factors, including TCR recombination^44–46^ and variant MHC alleles^47^, contributed to the patient-specific nature of TCR repertoire.

### Intra-patient similarity of TCR repertoire within multiple biopsies

While we were looking at sample-wise similarity, we found that similarity within multiple biopsies (e.g. tissues of multiple regions or PBMCs) from same patient were consistently high in many patients (Fig. 1C). Specifically, this intra-patient similarity was observed by the formation of patient-specific cluster, which was found in 6 out of the 9 PBMC samples (excluding OV12-PBMC, OV18-PBMC, EM5-PBMC) and all 31 tissue samples. To focus on intra-patient similarity, we re-organized similarity data based on each patient with metastatic cancer (Fig. 1C). The six representative insets show marked similarity constituting clusters of three or more samples. The maximum similarity of TCR repertoires occurred between tissues from the same organ within a single patient (Omentum1 and Omentum2 from patient “OV30”, Jaccard index = 0.273) but similarity across metastatic organs were also high compared to inter-patient similarity.

Interestingly, a particular patient labeled “OV30”, who underwent surgery twice so that two timepoints were investigated, showed similarity between samples at two different timepoints (Fig. 1C). Specifically, within the 1st timepoint samples, the average similarity was 0.153, and within the 2nd timepoint samples, the average similarity was 0.128. Importantly, between the samples of the 1st and 2nd time points, the average similarity was 0.114, indicating a notable consistency in the tumor TCR repertoire across these timepoints. In patient labeled “OV31”, who also had surgery twice showed a maintenance of previous clones from tissue and PBMC samples, which supports our observation on patient “OV30” (Fig. S3). We thought that this result can be presumably explained in two ways. One possibility is that tissue-resident memory T cells were expanded across adjacent tumor regions. The other possibility is that circulating memory lymphocytes infiltrated into tumor tissue.

In patients with ovarian cancer, we compared the number of unique CDR3 segments and Shannon’s entropy to assess the level of diversity within TCR repertoires. A high value of Shannon’s entropy corresponds to a high diversity in the distribution of TCR clones, which means that the TCR repertoire is likely to contain high proportion of low frequency TCR clones^48^. Both metrics in PBMC samples tended to be greater than that in tumor samples (Fig. S4). This is in accordance with previous results showing a relatively low number of unique CDR3 sequences in tissues compared with PBMCs^49–51^.

The data in Fig. 1C suggest that the overlap in TCR repertoires between PBMCs and TILs is higher within individual donors than between donors. We plotted the overlap in TCR repertoires between PBMCs and TILs (Fig. 2A,B) and observed that samples from the same patient tended to overlap more with each other than with samples from different patients (Fig. 2C). Furthermore, the results from patients “OV30” and “OV31” showed that overlap of TCR clones between PBMCs and TILs could be detected at different times within the same patients (Fig. 2A).

### The PBMC TCR repertoire has high overlap with clones found in multiple tissue sites

We next focused on TCR clones overlapping in multiple organs. Since the similarity between samples (Figs. 1C and 2A,B) is a value calculated between two samples, there is a limitation in that it cannot explain the overlap with multiple organs as well. Thus, we pooled all of the TIL TCR clones within the same surgical case and grouped the unique TIL TCR clones by the number of tissues they are observed (Fig. 3A,B). Among the TCR clones found in multiple tissues, relatively high proportion of PBMC TCR clones were found compared to TCR clones found in single tissue. We then performed a Chi-square test to determine whether the increased proportion of PBMC TCR clones in a group of multiple tissue-derived TCR clones was statistically significant (Supplementary Tables S2 and S3). The resulting P values for nine cases converged to near zero, indicating that TCR clones found in multiple tissue samples are jointly detected in the PBMC TCR repertoire.

### Multiple tissue sites share clonally abundant TCR clusters with CDR3 sequence similarity

**Figure 4:**
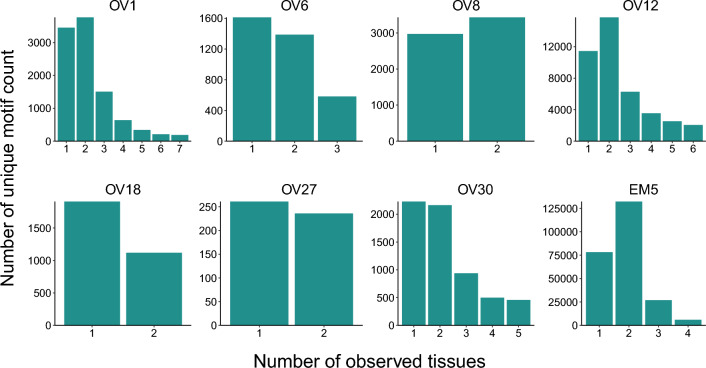
TCR motifs are shared among multiple metastatic tissues. Unique TCR motifs within eight patients with metastatic cancer were grouped. The number of tissues in which each motif occurred was then counted. For example, a total of five tissue samples in “OV30-1st” and “OV30-2nd” shared the same TCR motifs (“OV30-1st” and “OV30-2nd” were from a single patient that underwent surgery twice).

Because TCR clones can have extensive diversity due to the complexity of combinations in their variable domains, we next focused on motifs or decomposed structural units of amino acid sequence. We used GLIPH2^36^ to cluster the CDR3 motifs by frequency and sequence similarity within eight patients with metastatic cancer. All eight patients had unique CDR3 motifs that were shared among multiple tissues with metastasis (Fig. 4). The sample clustering based on individual motif frequencies showed that some TCR clusters were conserved among multiple tissues with metastasis, which might be the result of a tumor antigen response (Fig. S5).

To indirectly determine whether tumor antigens from multiple tissue sites within the same patient were shared, we profiled variants in tumor tissues using a target-enrichment method (Fig. S6). We observed a total of 34 unique variants among the eight patients and found that 23 variants were shared among more than two tissues within the same patient. Although the total number of variants was not large, the proportion of variants that were shared among tissues was high. Therefore, we can infer that there was a high probability of shared neoantigens between metastasized tissues within the same patient.

## Discussion

Gynecologic cancer is difficult to treat, because most patients are diagnosed at an advanced stage and experience recurrence despite surgery and chemotherapy^52,53^. Recently, various types of cancer immunotherapy have been developed, improving treatment outcomes^54–56^. However, the current response rate for immunotherapy in patients with gynecologic cancer is only ~ 20%^57,58^, and predictive biomarkers have been explored to improve immunotherapy efficacy and enable personalized targeted therapy. The presence of TILs in the tumor microenvironment is associated with improved patient survival. In this study, we observed TCR clones that were highly conserved among multiple sites of metastasis, suggesting the presence of tumor-specific T cells that might be harnessed for immunotherapy in patients with gynecologic cancer.

We profiled the CDR3 sequences of patients with gynecologic cancer to measure inter-sample similarity in TCR repertoires. The elucidation of the TIL CDR3 sequences and the extent to which it is shared in multiple metastatic tissues may lead to finding the novel TCR-based therapeutics. High clonal similarity between TIL TCR repertoires is supported by other studies that showed homogeneity of TCR repertoires within organs^59^ and tissues^60^. Our data suggest that tumor samples from distant tissues share TCR clones, which might have expanded from similarly structured antigens.

Neoantigens shared among tumor tissues with high immunogenic potential may drive the clonal evolution of immune cells. Studies have shown that metastatic clones with the same origin share antigen variants^61,62^, and computationally predicted neoantigens were highly shared across metastatic sites^59,63^. Similarity of neoantigen pools between sites that are located closely together has not been explicitly shown, however. Hence, further study is needed to elucidate the neoantigens that are shared in tumor tissues.

We found that the TCR repertoire of PBMCs is relatively rich with clones that are shared by multiple metastases rather than clones that appear only at a single tumor site (Fig. 3A,B). The presence of tissue-shared clones in PBMCs was reported in a previous study^64^; however, that study contrasts with ours in that it compared TCR repertoires of tumor and non-tumor tissues with inflammation. In our understanding, recirculation of T cells is responsible for the high proportion of tissue-shared clones in blood^65^. However, we cannot exclude the possibility that the high proportion of tissue-shared clones might include bystander T cells^66^, which could potentially lessen the anti-tumorigenic potential, as we did not confirm whether the TCR sequences originated from tumor-specific T cells. Whether these clones function in tumor surveillance has not yet been studied.

We did not attempt to identify the sites where TCR repertoire expansion occurred. There remains a possibility that high-frequency TCR clones might have moved from the blood to tissues, which opposes our assumption that TIL TCR clones are generated from neoantigens in tumor tissues. In the work of Liangtao et al.^67^, tumor-specific TCR clones were verified by the RNA expression of proliferation-related markers. Furthermore, the fact that we did not perform experiments from Liangtao et al.^67^ is a weakness of our study. We believe that our analysis of the TCR repertoire overlap between tissues can be supplemented with sequencing data obtained through other experimental methods.

In recent years, several studies have aimed to understand the TCR repertoire similarities among patients with different types of cancers, with some delving into multi-omics approaches to provide a broader perspective, though not placing a primary focus on TCR similarity as our study did^68,69^. Our research distinctly stands on the data derived from Korean patients, shedding light on the TCR repertoire in gynecologic cancers such as ovarian and endometrial cancer. This unique ethnic dimension adds a novel premise to the existing body of knowledge, as different genetic backgrounds can significantly impact the immune response and, consequently, the TCR repertoire. Moreover, unlike some studies that did not utilize replicates^70–72^, our approach of employing only the clones common between replicates aimed to enhance the reliability of our findings. This approach brings a higher degree of confidence in the observed TCR repertoire similarities (Fig. S2), thereby providing a more robust basis for our conclusions. While there's an overlap in the principal goal of exploring TCR repertoire similarity with other studies, our study diverges through the adoption of a methodological approach and the utilization of a unique dataset, which allowed us to focus more intensively on this aspect. Our work, therefore, contributes a unique lens through which the TCR repertoire in gynecologic cancers can be understood, and presents a stepping stone for more generalized or diversified studies in the future.

In summary, we found TCR clones that were highly conserved between tissue sites, which may be a result of shared neoantigens expressed in the tissue environment. These tissue-shared clones were enriched in the TCR repertoire of PBMCs. Further collection of data is necessary to uncover the underlying biology of the immune microenvironment of gynecologic cancer.

### Supplementary Information

Supplementary Information 1.

Supplementary Information 2.

Supplementary Information 3.

## Data Availability

The sequencing data for tissue and PBMC TCR repertoire samples from gynecologic cancer patients have been deposited at the Sequence Read Archive under accession number PRJNA939934.
