# Supplementary material for: Globally shared TCR repertoires within the tumor-infiltrating lymphocytes of patients with metastatic gynecologic cancer
Source: Sci Rep. 2023 Nov 22;13:20485. doi: 10.1038/s41598-023-47740-2 (PMC10665396; doi:10.1038/s41598-023-47740-2)
Supplement: Supplementary file 1 — Supplementary Information 1. [file 41598_2023_47740_MOESM1_ESM.docx]

Supplementary Material

# Supplementary Figures

**Figure S1. Comparison of average frequency and abundance between twice-observed and once-observed CDR3 sequences. The frequency refers to the proportion of reads from an individual clone relative to the total reads in the group, calculated by dividing the number of reads from a unique clone by the total reads. The average frequency represents the mean frequency value for all clones, which is determined by summing the frequencies of all clones and dividing by the total number of clones, under the assumption that each clone has an equal frequency. The abundance is the cumulative sum of the frequencies of all clones.** Boxplots showing the average frequency and abundance of CDR3 sequences per sample (see Methods). Replicates were separated into two groups (once-observed and twice-observed). Ovarian cancer patient’s (A) average frequency, (B) abundance. Endometrial cancer patient’s (C) average frequency, (D) abundance. Wilcoxon signed-rank test, ns>=0.05, ${}^{***}p$<0.005.


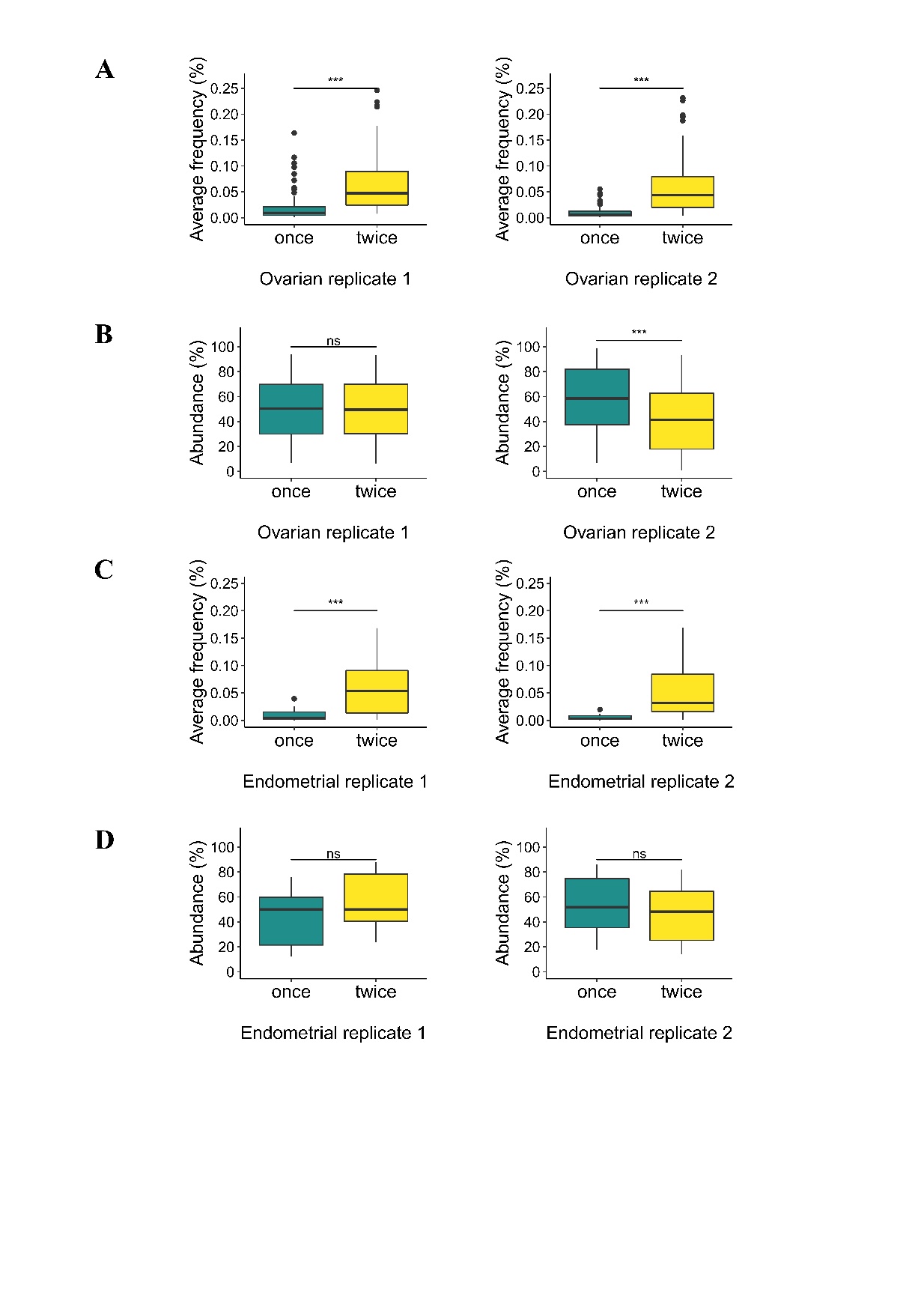


**Figure S2. Validation of replicate experiments.** The fraction of twice-observed unique CDR3 in (A) Ovarian PBMC, (B) Ovarian tissue, (E) Endometrial PBMC, (F) Endometrial tissue. The frequencies of CDR3s in each replicate shown in (C) Ovarian PBMC, (D) Ovarian tissue, (G) Endometrial PBMC, (H) Endometrial tissue. The fraction of twice-observed unique CDR3s in each replicate is calculated by dividing the number of twice-observed unique CDR3s by the total number of unique CDR3s in each replicate. Correlation of fraction of twice-observed unique CDR3s in each replicate (Ovarian PBMC: n=33, R= 0.8378191; Ovarian tissue: n=52, R= 0.6384844; Endometrial PBMC: n=8, R= 0.428595; Endometrial tissue: n=11, R= 0.6766915). Correlation of frequencies in each replicate (Ovarian PBMC: n=36,205, R=0.9513526; Ovarian tissue: n=76,441, R=0.7642012; Endometrial PBMC: n=12,567, R=0.9190504; Endometrial tissue: n=69,277, R=0.9368175).


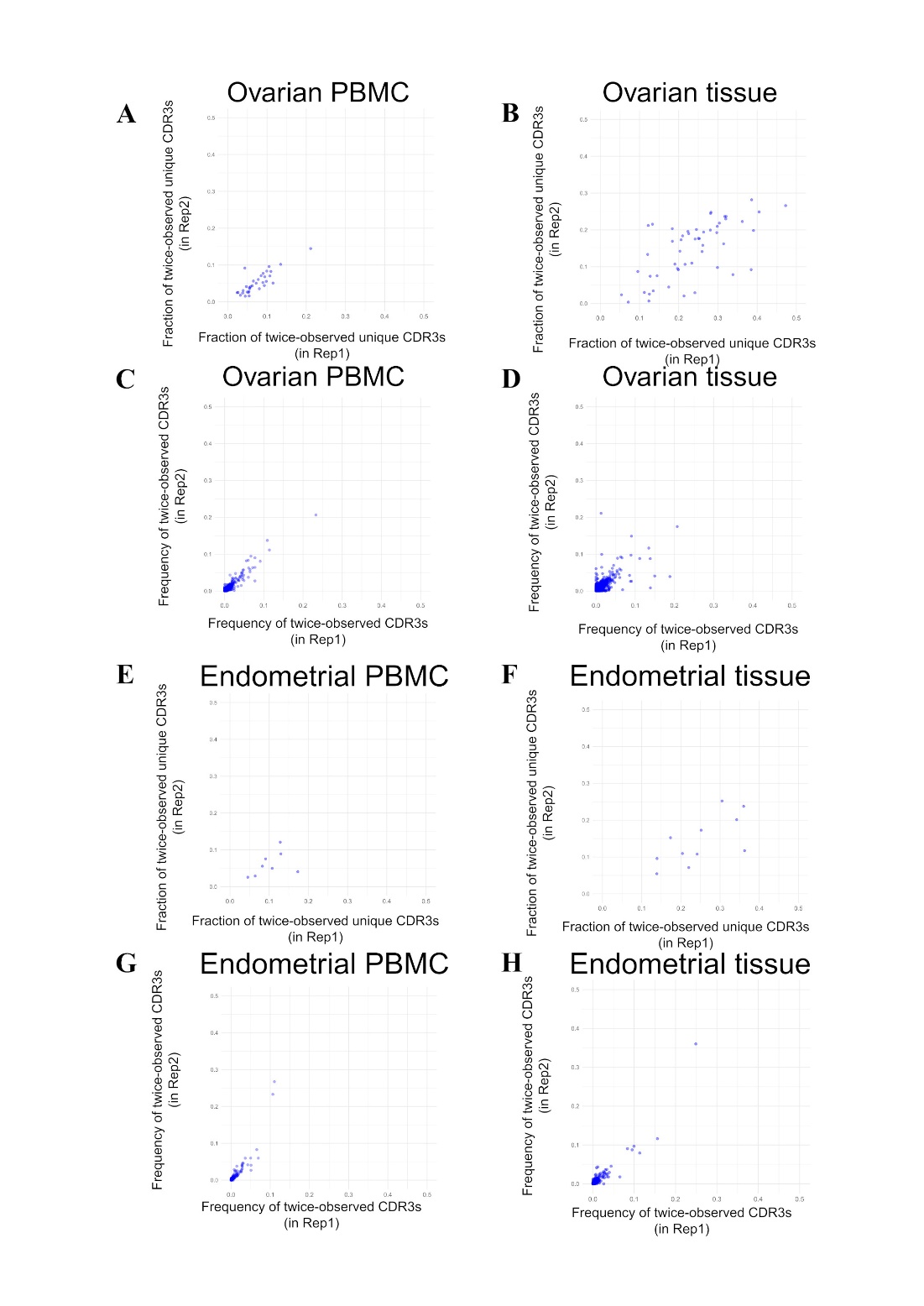


**Figure S3. Inter-sample overlap within patient OV31.** The heatmap shows the pairwise Jaccard index within the tissue and PBMC samples of patient OV31, who had surgery twice.


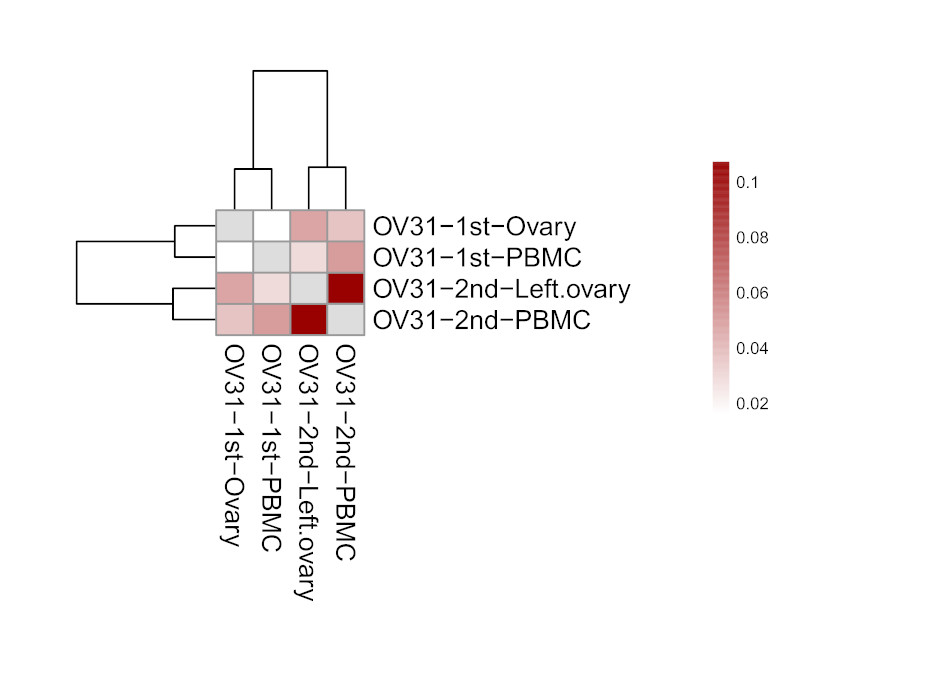


**Figure S4. Comparison of unique CDR3 sequences and diversity between PBMC and tissue.** Boxplots comparing (A) the number of unique CDR3 amino acid sequences (nCDR3) and (B) Shannon’s entropy between PBMC and tissue of ovarian cancer patients. Boxplots of endometrial cancer patients comparing (C) nCDR3 and (D) Shannon’s entropy is also shown. Each value was averaged within the replicates. Wilcoxon rank-sum test, ns>=0.05, ${}^{*}p$<0.05, ${}^{**}p$<0.01, ${}^{***}p$<0.005.


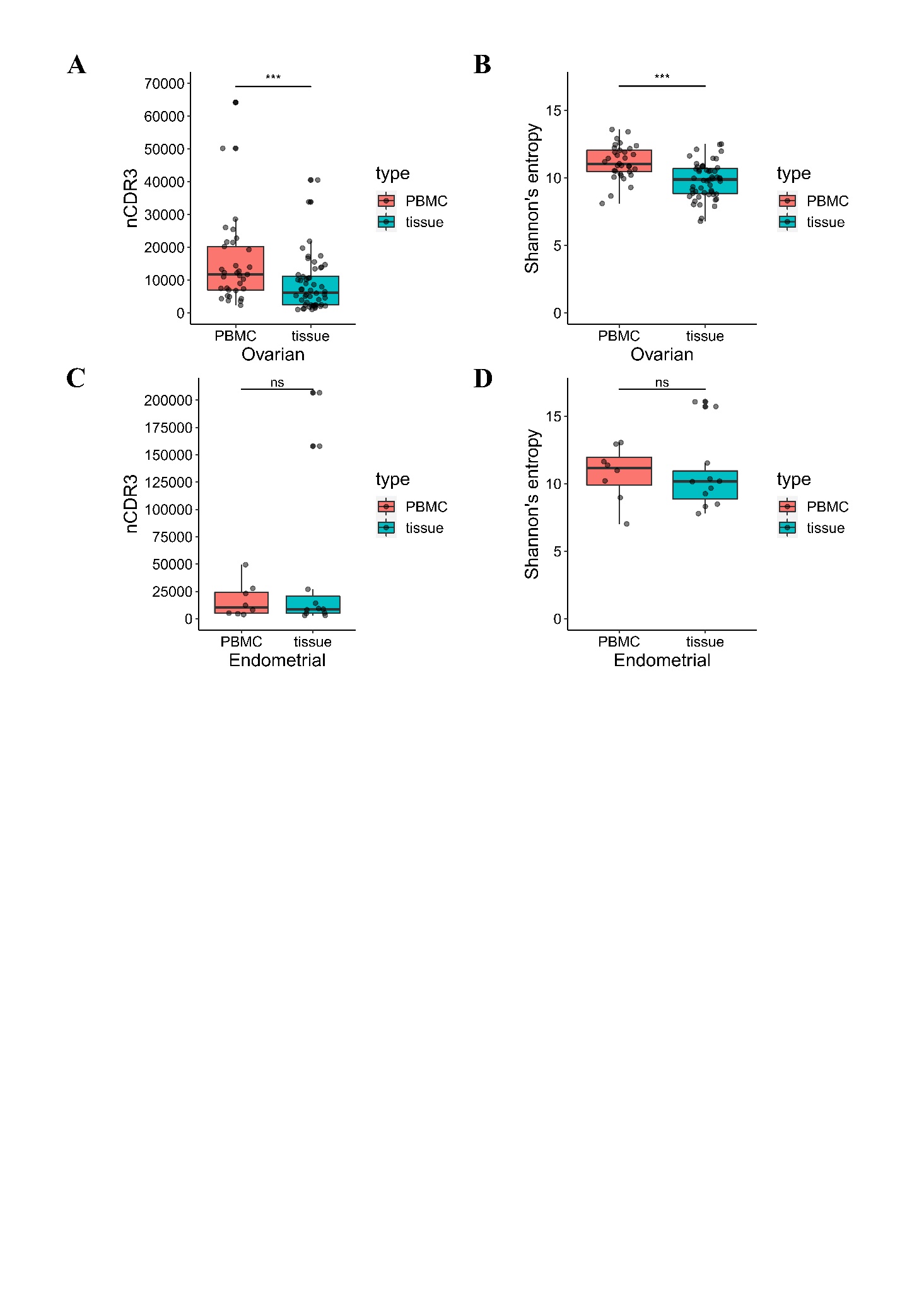


**Figure S5. Motif clustering in eight patients with metastatic cancer.** Heatmaps show motif frequencies within patients: (A) OV1, (B) OV6, (C) OV8, (D) OV12, (E) OV18, (F) OV27, (G) OV30, and (H) EM5. Motif clusters with Fisher score < 0.05 were selected. The value of each motif frequency was binarized as “Below mean”(< the mean motif frequency) or “Above mean”(≥ the mean motif frequency).


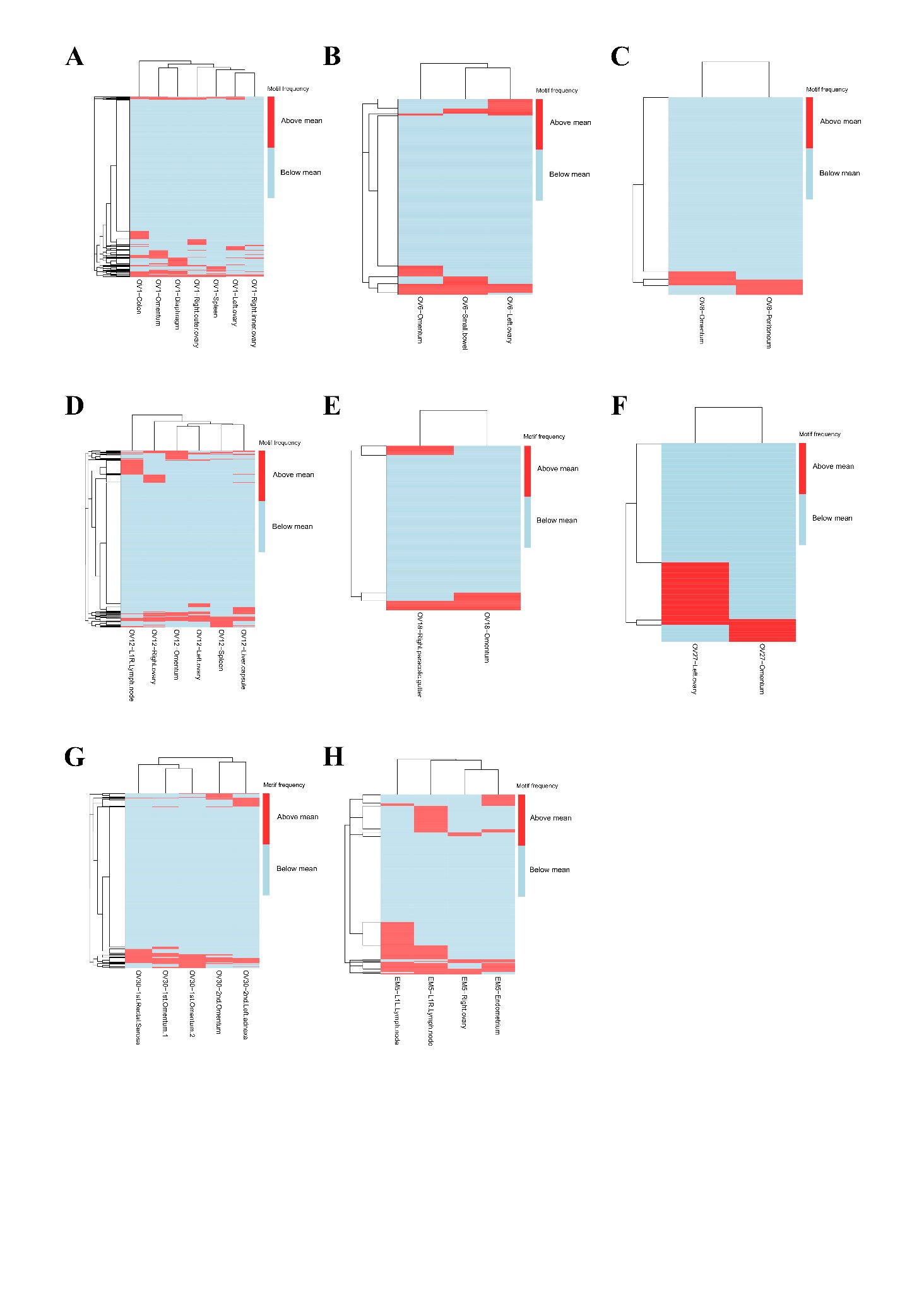


**Figure S6. Profiling of tumor tissue variants.** The allele frequencies of somatic variants in different tumor tissues were plotted in heatmaps.


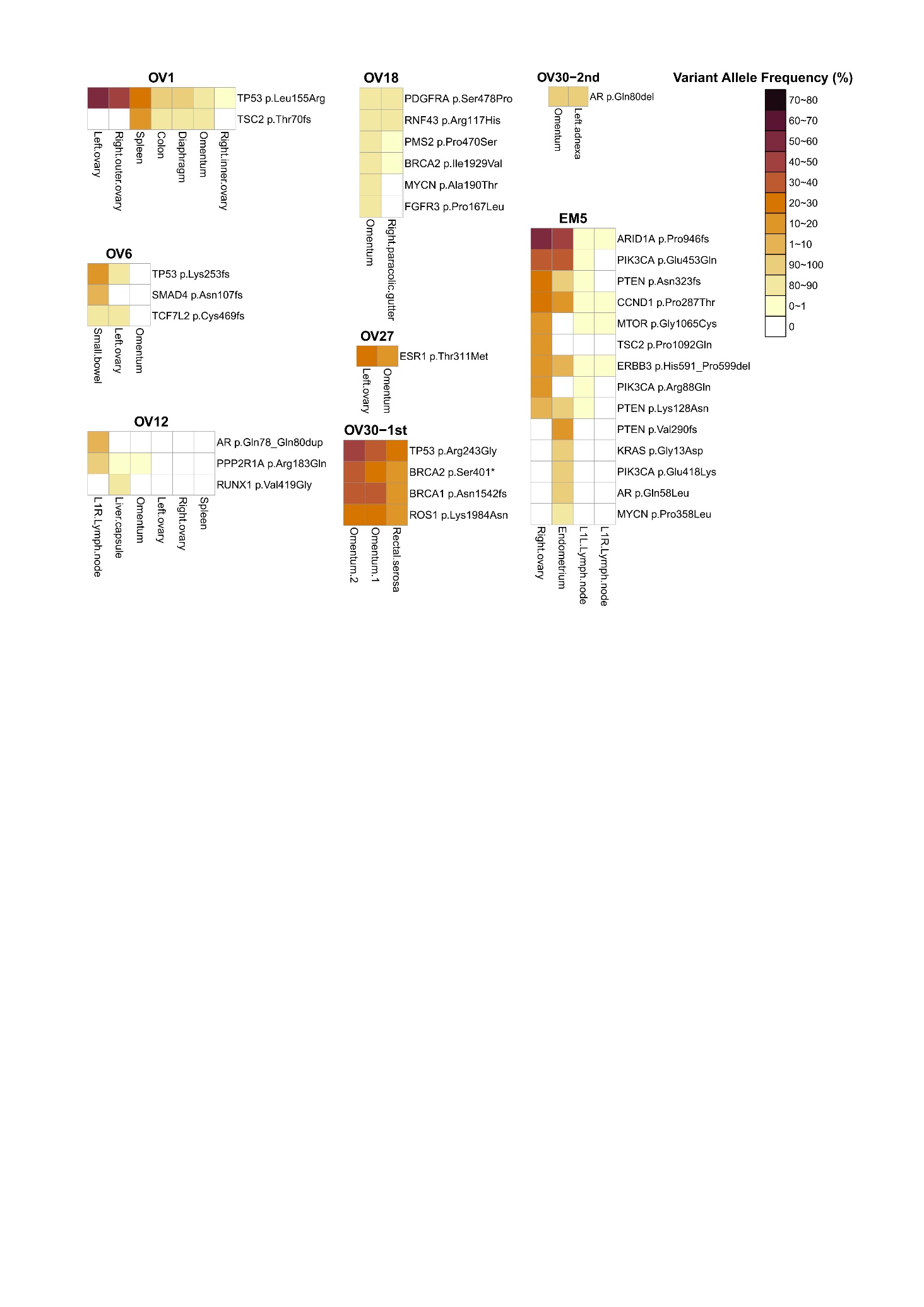


# Supplementary Tables

**Supplementary Table S1. Patient characteristics**

|  | All  (N = 39) | Ovarian cancer  (N = 31) | Endometrial cancer  (N = 8) |
| --- | --- | --- | --- |
| Age | | | |
| Median (range) | 57 (17–86) | 57 (17–86) | 62.5 (48–77) |
| Sex | | | |
| Female | 39 (100%) | 31 (100%) | 8 (100%) |
| Number of biopsy sites per patient | | | |
| 1 | 30 (76.9%) | 23 (74.2%) | 7 (87.5%) |
| 2 | 5 (12.8%) | 5 (16.1%) | 0 (0%) |
| ≥3 | 4 (10.3%) | 3 (9.7%) | 1 (12.5%) |
| Stage | | | |
| I | 3 (7.7%) | 1 (3.2%) | 2 (25%) |
| II | 0 (0%) | 0 (0%) | 0 (0%) |
| III | 13 (33.3%) | 10 (32.3%) | 3 (37.5%) |
| IV | 19 (48.7%) | 16 (51.6%) | 3 (37.5%) |
| Unknown | 4 (10.3%) | 4 (12.9%) | 0 (0%) |

**Supplementary Table S2. Chi-square test of the tendency of TIL clones to overlap with PBMCs or other tissue repertoires, performed on patients with multiple tissue samples**

| Tissue clones | Not detected in PBMCs | Detected in PBMCs |
| --- | --- | --- |
| Detected in 1 tissue | A | B |
| Detected in >1 tissue | C | D |

| Patient | Degrees of freedom | Chi-square | p-value |
| --- | --- | --- | --- |
| OV1 | 1 | 4004.5171 | 0 |
| OV6 | 1 | 399.01633 | 9.02E-89 |
| OV8 | 1 | 398.42842 | 1.21E-88 |
| OV12 | 1 | 413.72635 | 5.66E-92 |
| OV18 | 1 | 671.26273 | 5.31E-148 |
| OV27 | 1 | 82.111869 | 1.29E-19 |
| OV30-1st | 1 | 737.9116 | 1.71E-162 |
| OV30-2nd | 1 | 856.57623 | 2.70E-188 |
| EM5 | 1 | 2769.9145 | 0 |
| Metastatic | 1 | 10752.95 | 0 |

**Supplementary Table S3. List of tissue biopsy regions from patients with multiple tissue samples**

| Patient label | Tissue region |
| --- | --- |
| OV1 | Right inner ovary |
|  | Right outer ovary |
|  | Left ovary |
|  | Colon |
|  | Spleen |
|  | Diaphragm |
|  | Omentum |
| OV6 | Left ovary |
|  | Omentum |
|  | Small bowel |
| OV8 | Omentum |
|  | Peritoneum |
| OV12 | Left ovary |
|  | Right ovary |
|  | Omentum |
|  | Spleen |
|  | L1R Lymph node (External iliac node) |
|  | Liver capsule |
| OV18 | Omentum |
|  | Right paracolic gutter |
| OV27 | Left ovary |
|  | Omentum |
| OV30-1st | Omentum 1 (Different locations within omentum) |
|  | Omentum 2 (Different locations within omentum) |
|  | Rectal Serosa |
| OV30-2nd | Left adnexa |
|  | Omentum |
| EM5 | Right ovary |
|  | Endometrium |
|  | L1L Lymph node (External iliac node) |
|  | L1R Lymph node (External iliac node) |

**Supplementary Table S4. Primer design**

| 1st PCR: forward primers | |
| --- | --- |
| ID | Primer sequence (5’ to 3’) |
| TRBV2 | TGCTAGACTCAAGTGATCCANNNNNNGAAATATTCGATGATCAATTCTCAGTTGA |
| TRBV3-1 | TGCTAGACTCAAGTGATCCANNNNNNTTCTCACCTAAATCTCCAGACAAAGCT |
| TRBV4-1 | TGCTAGACTCAAGTGATCCANNNNNNTAAATGAAAGTGTGCCAAGTCGCTT |
| TRBV4-2 | TGCTAGACTCAAGTGATCCANNNNNNCTGAAAACAACAGTGTGCCAAGTC |
| TRBV5-1 | TGCTAGACTCAAGTGATCCANNNNNNAAACTTCCCTGGTCGATTCTCAGG |
| TRBV5-4 | TGCTAGACTCAAGTGATCCANNNNNNTTCTCAGGTCTCCAGTTCCCTAATTATAG |
| TRBV5-5 | TGCTAGACTCAAGTGATCCANNNNNNAGGAAACTTCCCTGATCGATTCTCAG |
| TRBV5-6 | TGCTAGACTCAAGTGATCCANNNNNNATTCTCAGGTCACCAGTTCCCTAACTATA |
| TRBV5-8 | TGCTAGACTCAAGTGATCCANNNNNNATTTTCAGGTCGCCAGTTCCCTAATTATA |
| TRBV6-1 | TGCTAGACTCAAGTGATCCANNNNNNCTACAATGTCTCCAGATTAAACAAACGGG |
| TRBV6-2_3 | TGCTAGACTCAAGTGATCCANNNNNNGTCTCCAGATTAAAAAAACAGAATTTCCT |
| TRBV6-4 | TGCTAGACTCAAGTGATCCANNNNNNTATAGTGTCTCCAGAGCAAACACAGATG |
| TRBV6-5 | TGCTAGACTCAAGTGATCCANNNNNNTACAATGTCTCCAGATCAACCACAGAG |
| TRBV6-6 | TGCTAGACTCAAGTGATCCANNNNNNATAAAGGAGAAGTCCCGAATGGCTAC |
| TRBV6-8 | TGCTAGACTCAAGTGATCCANNNNNNGTCTCTAGATTAAACACAGAGGATTTCCC |
| TRBV6-9 | TGCTAGACTCAAGTGATCCANNNNNNATGTATCCAGATCAAACACAGAGGATTTC |
| TRBV7-2 | TGCTAGACTCAAGTGATCCANNNNNNAGTGATCGCTTCTCTGCAGAGAG |
| TRBV7-3 | TGCTAGACTCAAGTGATCCANNNNNNCAAAGATCGGTTCTTTGCAGTCAGG |
| TRBV7-4 | TGCTAGACTCAAGTGATCCANNNNNNAACGAGACAAATCAGGGCGG |
| TRBV7-6 | TGCTAGACTCAAGTGATCCANNNNNNAATGATCGGTTCTCTGCAGAGAGG |
| TRBV7-8 | TGCTAGACTCAAGTGATCCANNNNNNAGTGATCGCTTCTTTGCAGAAAGG |
| TRBV7-9 | TGCTAGACTCAAGTGATCCANNNNNNGAAAAATCAGGGCTGCTCAGTGATC |
| TRBV9 | TGCTAGACTCAAGTGATCCANNNNNNAAGGAAACATTCTTGAACGATTCTCCG |
| TRBV10-1 | TGCTAGACTCAAGTGATCCANNNNNNTAACAAAGGAGAAGTCTCAGATGGCTAC |
| TRBV10-2 | TGCTAGACTCAAGTGATCCANNNNNNTATTACAGATAAAGGAGAAGTCCCCGATG |
| TRBV10-3 | TGCTAGACTCAAGTGATCCANNNNNNCAGATGGCTATAGTGTCTCTAGATCAAAG |
| TRBV11-3 | TGCTAGACTCAAGTGATCCANNNNNNCACAGTTGCCTAAGGATCGATTTTCTG |
| TRBV12-3 | TGCTAGACTCAAGTGATCCANNNNNNCTAAGATGCCTAATGCATCATTCTCCAC |
| TRBV12-4 | TGCTAGACTCAAGTGATCCANNNNNNCAGCTAAGATGCCTAATGCATCATTCTC |
| TRBV12-5 | TGCTAGACTCAAGTGATCCANNNNNNCAGAGATGCCTGATGCAACTTTAGC |
| TRBV13 | TGCTAGACTCAAGTGATCCANNNNNNCTCAGCTCAACAGTTCAGTGACTATCAT |
| TRBV14 | TGCTAGACTCAAGTGATCCANNNNNNCAACAATCGATTCTTAGCTGAAAGGACTG |
| TRBV15 | TGCTAGACTCAAGTGATCCANNNNNNAATGAAGCAGACACCCCTGATAACTT |
| TRBV16 | TGCTAGACTCAAGTGATCCANNNNNNGTATGCCCAAGGAAAGATTTTCAGCTAAG |
| TRBV18 | TGCTAGACTCAAGTGATCCANNNNNNACGATTTTCTGCTGAATTTCCCAAAGAG |
| TRBV19 | TGCTAGACTCAAGTGATCCANNNNNNAAAGGAGATATAGCTGAAGGGTACAGC |
| TRBV20-1 | TGCTAGACTCAAGTGATCCANNNNNNGAGAAGGACAAGTTTCTCATCAACCATG |
| TRBV24-1 | TGCTAGACTCAAGTGATCCANNNNNNGAGATCTCTGATGGATACAGTGTCTCTC |
| TRBV25-1 | TGCTAGACTCAAGTGATCCANNNNNNCTGAGTCAACAGTCTCCAGAATAAGGAC |
| TRBV27 | TGCTAGACTCAAGTGATCCANNNNNNAGTCTCTCGAAAAGAGAAGAGGAATTTCC |
| TRBV28 | TGCTAGACTCAAGTGATCCANNNNNNGAAAAAGGAGATATTCCTGAGGGGTACAG |
| TRBV29-1 | TGCTAGACTCAAGTGATCCANNNNNNGAGAGTGGATTTGTCATTGACAAGTTTCC |
| TRBV30 | TGCTAGACTCAAGTGATCCANNNNNNATTGACCAGATCAGCTCTGAGGTG |

| 1st PCR: reverse primers | |
| --- | --- |
| ID | Primer sequence (5’ to 3’) |
| TRBJ1-1 | GCGATGACGAACTTCGTTTCNNNNNNTGGTGCCTTGTCCAAAGAAA |
| TRBJ1-2 | GCGATGACGAACTTCGTTTCNNNNNNGTCCCCGAACCGAAGGTG |
| TRBJ1-3 | GCGATGACGAACTTCGTTTCNNNNNNGCCAACTTCCCTCTCCAAAATAT |
| TRBJ1-4 | GCGATGACGAACTTCGTTTCNNNNNNGGGTTCCACTGCCAAAAAAC |
| TRBJ1-5 | GCGATGACGAACTTCGTTTCNNNNNNGAGTCCCATCACCAAAATGC |
| TRBJ1-6 | GCGATGACGAACTTCGTTTCNNNNNNGTCCCGTTCCCAAAGTGG |
| TRBJ2-1 | GCGATGACGAACTTCGTTTCNNNNNNGTCCCTGGCCCGAAGAAC |
| TRBJ2-2 | GCGATGACGAACTTCGTTTCNNNNNNCCTAGAGCCTTCTCCAAAAAAC |
| TRBJ2-3 | GCGATGACGAACTTCGTTTCNNNNNNGTGCCTGGGCCAAAATAC |
| TRBJ2-4 | GCGATGACGAACTTCGTTTCNNNNNNCACTGAGAGCCGGGTCCC |
| TRBJ2-5 | GCGATGACGAACTTCGTTTCNNNNNNGTGCCTGGCCCGAAGTAC |
| TRBJ2-6 | GCGATGACGAACTTCGTTTCNNNNNNAGCACGGTCAGCCTGCTG |
| TRBJ2-7 | GCGATGACGAACTTCGTTTCNNNNNNTGACCGTGAGCCTGGTGC |

| 2nd PCR: forward and reverse primers | |
| --- | --- |
| ID | Primer sequence (5’ to 3’) |
| V_2nd | ACACTCTTTCCCTACACGACGCTCTTCCGATCTTGCTAGACTCAAGTGATCCA |
| J_2nd | GTGACTGGAGTTCAGACGTGTGCTCTTCCGATCTGCGATGACGAACTTCGTTTC |
